# Factors Associated with Free Hospital Outpatient Service Use among Middle-Aged and Older Urban Homeless Adults in Taipei

**DOI:** 10.3390/ijerph18105330

**Published:** 2021-05-17

**Authors:** Lan-Ping Lin, Li-Yun Wang, Tai-Wen Wang, Yun-Cheng Chen, Jin-Ding Lin

**Affiliations:** 1 Department of Senior Citizen Care and Welfare, Ching Kuo Institute of Management and Health, Keelung 203, Taiwan; lanping518@gmail.com; 2 Department of Family Studies and Child Development, Shih Chien University, Taipei 104, Taiwan; nose.123@yahoo.com.tw; 3 School of Public Health, National Defense Medical Center, Taipei 114, Taiwan; s8038129@gmail.com (T.-W.W.); 607810031@mail.ndmctsgh.edu.tw (Y.-C.C.); 4 Institute of Long-Term Care, Mackay Medical College, New Taipei City 252, Taiwan

**Keywords:** homeless, outpatient, healthcare utilization, Andersen model

## Abstract

Homeless individuals have many negative experiences with inequality regarding access to and the use of primary healthcare services, so policies to eliminate the disparities in and barriers to primary care access for these people are needed. The aim of this study was to explore the use and determinants of free hospital outpatient services for homeless people, in order to describe the provision of free healthcare policies for this vulnerable population in Taipei. One cross-sectional survey was conducted to recruit homeless people aged 45 years old and over in Taipei in 2018. A structured questionnaire was used, and face-to-face interviews were conducted by three social workers to collect the data. Finally, 129 participants were recruited in the study. The results show that 81.4% of the homeless people had made free hospital outpatient care visits (mean = 5.9 visits) in the last three months. An unadjusted logistic regression analysis showed that those homeless people who reported having usual healthcare providers, with higher depressive symptom scores, who used medication and had been hospitalized within one year, and had more chronic diseases, were significantly more likely to make free hospital outpatient visits. The adjusted logistic regression model indicates that homeless people with severe depressive symptoms (odds ratio (OR) = 9.32, 95% CI = 1.15–56.07), who had received medication (OR = 3.93; 95% CI = 1.06–14.52), and who had more than five chronic diseases (OR = 1.06, 95% CI = 1.35–13.27), were significantly more likely to make free hospital outpatient visits than their counterparts. The findings highlight that homeless people have higher healthcare requirements than the general population, and the healthcare system should pay more attention to factors associated with higher outpatient service use, such as homelessness, severe depressive symptoms, the receipt of medication and chronic diseases.

## 1. Introduction

Homelessness is a major public health problem that requires special attention in society [1]. As the population of older adults increases in many countries, there is a parallel increase in the numbers of older adults who are experiencing homelessness [2]. Homelessness is associated with an increase in medical use and cost. There is a need for public health and policy efforts that support healthcare systems for homeless persons, so as to reduce disparities in primary care accessibility and improve health outcomes for this population [3]. In a nationally representative survey in the United States, homeless persons reported severe barriers to needed care and used acute hospital-based care at high rates [4]. Compared with the general population, homeless people report worse mental health, and thus represent a particularly vulnerable group. Moreover, they have more poor health habits, they suffer from chronic conditions and they tend to access emergency rooms more frequently [5]. In addition, homeless adults have high rates of functional vision impairment, as well as skin, leg and foot problems [6]. As a consequence, they have an increased risk for premature mortality [1].

According to a study by the Boston Healthcare for the Homeless Program [7], the burden of disease is high, with the majority of patients experiencing mental illness, substance use disorders and a number of diseases. Hospitalization and emergency room use were shown to be frequent, and the total expenditure was 3.8 times that for an average Medicaid recipient. In Toronto, Canada, Hwang et al. [8] found that even with a universal health insurance system, homeless people make substantially more emergency department visits and use hospitals more than the general population. In European capital cities, Canavan et al. [9] found that mental health service provision is variable, reporting major and common barriers to service accessibility, such as prejudice in serving the homeless, a lack of co-ordination amongst services and the difficulties homeless people face in obtaining health insurance. According to the above, homeless people have much higher rates of morbidity, resulting from their worse access to healthcare services than the general population.

Cardiovascular disease is a major cause of death among homeless adults, with significant disparities in in-hospital care and mortality between homeless and nonhomeless adults with cardiovascular conditions [10]. Disease patterns among the homeless in Tokyo have been characterized by alcoholic psychosis and alcohol-dependence syndrome, liver disease, pulmonary tuberculosis, diabetes mellitus, fractures, dislocations, sprains and strains, hypertension and cerebrovascular disease [11]. Generally, homeless persons face four major barriers to care: financial, bureaucratic, programmatic and personal [12]. Although engagement with health services and adherence to treatments are often compromised, homeless people typically attend the emergency department more often than nonhomeless people [13]. These health problems and service difficulties met by the homeless in their daily lives require further exploration.

The total number of homeless persons in Taiwan in 2019 was 3040, of which 86.2% were men and 13.8% were women. Within this homeless population, 27.8% were living in shelters and 72.2% were living on the streets. In total, 80% were located in municipalities, with 821 homeless people (125 women and 697 men) located in Taipei [14]. According to The Investigation Report on the Living Conditions of the Homeless in Taipei City in 2016 [15], the most common welfare service measures used by the homeless people interviewed were the supply of goods and food, physical hygiene and grooming services, medical treatments and cash subsidies. The Taipei City government currently provides services such as the counseling, occupational and living rehabilitation programs, medical assistance and services, employment counseling, short-term and referred placement care and warm winter programs [16]. To help homeless people who are unable to seek medical treatment, the Taipei City Government’s Social Welfare Bureau established a Homelessness Health Checkup Clinic and Medical Billing Program to eliminate the financial barriers to accessing medical care. The program includes (1) a health check before the homeless people enter the service institution, (2) annual health check-ups for institutional homeless, (3) an outpatient and inpatient medical expenses waiver and (4) other related costs derived from medical needs. The largest expenditure items are outpatient and inpatient medical expenses; the funding was NTD 6.5 million in 2014 [15]. Homeless people registered in Taipei City, who are not covered by the National Health Insurance, are provided with subsidies in accordance with the relevant provisions of the Taipei City Citizens’ Medical Subsidy Schemes. In addition, during the hospitalization period, those who are medically recommended to hire a special caregiver are provided with a partial subsidy after evaluation by a social worker [16]. Medical institutions in Taiwan are currently divided into medical centers, regional hospitals and primary clinics according to the health and welfare categories. Hospitals at different levels have different care tasks and roles, particularly in providing specialized outpatient and inpatient care [17]. If the primary clinics are unable to determine the ailment of the patient or provide complete treatment due to limited personnel, equipment and expertise, they should recommend the patient to be referred [18]. However, given the free medical treatment in Taiwan, the promotion of referred medical care entails people seeking medical treatment as a result of changes in the healthcare behavior and service types of medical institutions [19]. This means the referral system is currently ineffective, and most patients seek medical care, including outpatient care, at the hospital directly. 

The Taiwan National Health Insurance program, adopted in March 1995, is compulsory for all citizens starting from birth. When people fall ill, the government uses the health insurance premiums it receives to help patients pay part of their medical and medication costs [20]. The characteristics of the Taiwanese system include good accessibility, comprehensive population coverage and short waiting times. One consequence of easy accessibility to specialists is that the “gatekeeper” role of family doctors is relatively weak in Taiwan [21]. Since most homeless people experience economic difficulties, and have no regular living arrangement, they are often withdrawn from the national health insurance program. In Taipei City, as homeless people have medical care needs, social workers assess their medical needs and can issue a free medical voucher, which allows them to use healthcare services for free. Two affiliated hospitals provide free medical care services for the homeless in Taipei City. Homeless people can attend these two hospitals to be assisted by the hospital’s social workers in arranging medical treatment, and the medical expenses are paid by the Social Welfare Bureau of Taipei City Government. The medical billing plan has greatly improved medical care for the homeless in Taipei City [15]. Few studies have measured user primary care utilization and determinants among homeless individuals in Taiwan, despite the high prevalence of diseases and disorders and the government’s policy of healthcare subsidies for this population. Older homeless adults have high rates of geriatric conditions, which may increase their chances of acute care use and nursing home placement [22]. The homeless elderly is the most vulnerable section of this impoverished population, and more research is needed to define their mental and physical health needs and to find ways of providing them with healthcare services [23]. The aim of this study was to explore the use and determinants of healthcare services in homeless people to facilitate the provision of the free healthcare for this vulnerable population in Taipei.

## 2. Methods

We explored free outpatient care use and its associated factors among urban middle-aged and elderly homeless people in Taipei. We used the Andersen’s behavioral model of health services use [24], which analyzes the usage of health services including inpatient care, physician visits and dental care, among others. It operates on multilevel dynamics, including predisposing factors, enabling factors and needs. This model has been used extensively in studies investigating the use of health services [25]. The present study adopted this model to examine predisposing factors (age, sex, educational level and marital status), enabling factors (personal total income, employment and regular medical provider) and need factors (physical and mental health and medication experience) in order to identify the factors of health service use among homeless people. We investigated depressive symptoms, based on the Patient Health Questionnaire-9 (PHQ-9), to examine respondents’ mental health. Depression severity was categorized into five levels (determined by the PHQ-9 score): none (0–4), mild (5–9), moderate (10–14), moderately severe (15–19), and severe (20–27).

In Taiwan, 80% of the homeless are located in urban areas. Taipei City has the greatest population of homeless people, with 647 homeless individuals listed by the services for homeless people in 2017 [26]. Wanhua District and Taipei Main Station had larger populations of homeless people than other areas in this city. Therefore, we employed a cross-sectional survey to recruit homeless people aged 45 years and over (*n* = 292) from these two areas. A structured questionnaire was used, and face-to-face interviews were conducted by three social workers to collect data from the following settings: the offices of the Wanhua District Welfare Center, Homeless Taiwan, homeless shelters, Taipei Main Station and Wanhua Park. Generally, expert surface validity was ensured in this study, and the reliability of the questionnaire was assessed using Cronbach’s alpha (0.865). Regarding ethical considerations, first, the main study locations (the Wanhua District Welfare Center and Homeless Taiwan) agreed to participate in this study after officially reviewing the research proposal. Second, we clarified the study’s purposes and right-protection protocols for the homeless individuals and obtained their informed consent prior to participation. 

The response data were analyzed using the SPSS software. The statistical method included describing the participants’ characteristics and their free outpatient visits, and then using univariate chi-square analyses to explore the relations between free hospital outpatient visits and the participants’ characteristics. We defined a high hospital outpatient care user as a homeless person who makes more than one visit per month (≥3 visits in three months). Finally, multiple logistic regression models were tested to identify the factors associated with high free hospital outpatient use among homeless adults.

## 3. Results 

### 3.1. Demographic Characteristics of the Study Participants 

In total, 129 homeless people participated in the study among the study population of 292. The demographic characteristics of the homeless people in this study are shown in Table 1. Their average age was 58.3 years, and 88.4% of the subjects were men. According to their educational background data, 27.9% had an elementary school education or less, 30.2% had a junior high school education, 33.3% had a senior high school education and 8.6% had a college education or above. Regarding their marital status, 51.2% reported being single, 36.4% were divorced, 41.1% reported being unemployed, 44.9% had part-time employment and only 14% had a full-time job. In terms of the source of living income, 55.1% expressed depending on their own earnings,11.6% and 3.1% reported using their disability and low-income allowance, respectively, 30.2% used other sources, such as family or charity help. Of the participants, 54% had a usual healthcare provider whom they would visit if sick or in need of advice about their health. Nearly half of the people with ID had multiple handicaps or disabilities. Table 1 also illustrates the depressive conditions among the participants. The results show that 15.5% were free from depression, 16.3% had mild depression, 31.8% had moderate depression, 26.4% had moderately severe depression and 10.1% had severe depression. Many of the homeless individuals needed to return to healthcare facilities for further checks, and age was associated with depressive symptoms among middle-aged and older homeless adults [18].

### 3.2. Hospital Outpatient Department Visits among the Participants 

**Figure 1:**
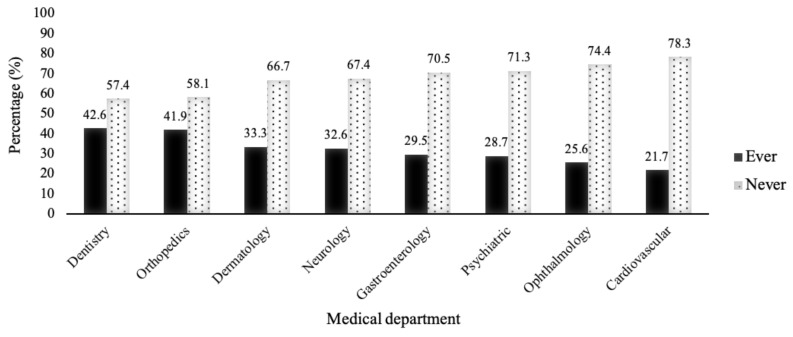
Distribution of medical department use in homeless people.

A total of 81.4% of the homeless people reported making free outpatient visits in the three months prior to the questionnaire being administered (Table 2). The mean number of hospital outpatient visits was 5.9. Of those who used free outpatient care, 58.1% reported fewer than three visits, while 41.9% made more than three visits in the period. Figure 1 shows the distribution of medical department use amongst homeless people. The most used medical departments were dental care, orthopedics, dermatology, neurology, gastroenterology, psychiatric, ophthalmology and cardiovascular. The reasons for medical department use by frequency were psychiatric (9.1 visits), hepatobiliary and gastroenterology (8.7 visits), dermatology (8.6 visits), orthopedics (8.5 visits), dental (7.9 visits), cardiovascular (6.3 visits), and neurology (5.7 visits) (Table 2).

### 3.3. Determinants of Free Outpatient Visits

Table 3 shows a one-way analysis of free hospital outpatient visits for two groups (low vs. high use) among the participants. One enabling factor amongst homeless people who had a usual healthcare provider, and who had needs factors of higher PHQ score and severity, receipt of medication and inpatient care within one year, made more hospital outpatient visits, and had more chronic diseases, was a statistical difference in free hospital outpatient visits. The other predisposing factors, such as sex, age, level of education, marital status and employment status, were not statistically different between groups with high and low free hospital outpatient visit frequency. To understand the factors associated with the expressed health needs of homeless people, Table 4 provides an analysis of the factors associated with free hospital outpatient visits among the homeless. The unadjusted logistic regression model shows the bivariate relationship between an independent and dependent variable, and it does not control for covariates or confounders. The results indicate that homeless people with usual healthcare providers (OR = 2.77, 95% CI = 1.33–5.77), with severe depression (mild, OR = 4.40, 95% CI = 1.10–17.68; moderately severe, OR = 4.50, 95% CI = 1.24–16.28; severe, OR = 13.33, 95% CI = 2.45–72.45), who receive medication (OR = 8.81, 95% CI = 2.88–26.92), who had used inpatient care within one year (OR = 2.78, 95% CI = 1.30–5.94), and those with more than five chronic diseases (OR = 10.18, 95% CI = 3.81–27.22), made significantly more free hospital outpatient visits than their counterparts. For the adjusted logistic regression model, which controls for the significant variables in the unadjusted analysis, the results indicate that homeless people with severe depression symptoms (OR = 9.32, 95% CI = 1.15–56.07), those who received medication (OR = 3.93; 95% CI = 1.06–14.52), and those with more than five chronic diseases (OR = 1.06, 95% CI = 1.35–13.27), were more significantly associated with a high frequency of free hospital outpatient visits than their counterparts. The results suggest that the need domain in Andersen’s model of healthcare use is the most significant factor determining the high usage of hospital outpatient care seen in this study. 

## 4. Discussion

Evidence on the management of diseases, and the long-term care, treatment and health promotion of homeless adults is sparse, but this information is required in order to provide healthcare, and to assess healthcare use and outcome effectiveness [27,28]. This study aimed to explore the use and associated factors of healthcare services in homeless people to assess the provision of free healthcare in this vulnerable population in Taipei. The results showed that 81.4% of homeless people made free hospital outpatient visits (mean = 5.9 visits) in the last three months, resulting in an estimated number of annual outpatient visits of nearly 24. This figure is higher than in the general population under the Taiwan National Health Insurance scheme (mean = 15.1 visits, which includes Western medicine, Chinese medicine, and dental care in 2017) in Taiwan [29]. However, the hospital outpatient care amongst the homeless only partially covers their expressed needs rather than covering their entire healthcare needs. Homeless people have greater difficulty in meeting their economic and general living needs, and would require more services than average, since needs drive service use. Furthermore, Nakonezny and Ojeda [30] found that the high level of outpatient use is probably because the homeless outreach program eliminates many of the barriers that prevent homeless individuals from receiving primary medical care. In a study in Québec, Canada, frequent users of public ambulatory health services were the most dissatisfied. Strategies for improving satisfaction include promoting primary care programs (including family physicians) that are more adapted to the needs of this population, and better integrating primary care within specialized services [31]. In this study, 54% of participants reported having a usual healthcare provider; however, almost half of the homeless still lacked usual providers to deliver accessible, comprehensive patient-centered care. In the future, we can evaluate the experience and satisfaction of homeless people regarding healthcare visits in order to monitor the free care system in place for this group of people. 

Amongst homeless people, the most-used medical departments were dental care (42.6%), orthopedics (41.9%), dermatology (33.3%), neurology (32.6%), gastroenterology (29.5%), psychiatric (28.7%), ophthalmology (25.6%) and cardiovascular (21.7%). These figures illustrate a high prevalence of high-burden medical conditions and high potential healthcare use. These homeless people are at relatively high risk for a broad range of acute and chronic illnesses. Certain illnesses and health problems may frequently be the antecedents of homelessness, and the quality of these people’s healthcare may need more attention. Jones et al. [32] found that a large cohort of homeless patients with community-acquired pneumonia exhibited a higher hospitalization risk than, but similar length of stay and costs to, nonhomeless patients. Homeless people display higher rates of premature mortality than the rest of the population, especially from suicide and unintentional injuries, and also suffer an increased prevalence of a range of infectious diseases, mental disorders and kinds of substance misuse [13]. In the United States, Wadhera et al. [33] analyzed the data from 2007 to 2013, and found that hospitalizations among homeless persons were increasing, and mental illness and substance use were the primary drivers of acute hospitalization among homeless adults. O’Toole et al. [34] concluded that health service use among the homeless is substantial and independently associated with sheltering arrangement, comorbid illness, health insurance and social support. Swabri et al. [35] explored and determined the specific reasons for attending mobile health units, and found high rates of physical and mental health conditions in homeless people. Infectious diseases such as hepatitis C (29%) and depression (43%) were prevalent, and dental disease was present in 79%. The present findings and recent research address dental and chronic diseases, mental illness and the related needs of the homeless, determining that they should keep in touch with hospitals to ensure that they follow through with their treatment plans.

Our one-way analysis results showed that homeless people with usual healthcare physicians, with severe depressive symptoms, who had received medication and inpatient care within one year, who made more outpatient visits, and who had more chronic diseases, had a significantly higher rate of free hospital outpatient visits. This means that the enabling and need factors are significantly associated with free hospital outpatient visits in the homeless. These findings are similar to those reported by Lindamer et al. [36], who found that the characteristics most strongly associated with heavy usage of the public mental health system were enabling and need factors. In a study in Dublin, Ireland, Keogh et al. [37] found that homeless individuals had higher usage rates of prescription medicines; however, they observed a decrease in attendance at outpatient departments and a decreasing trend in attendance at accident and emergency departments. Zuccaro et al. [38] also stated that current outpatient services may not meet the surgical care needs of these patients, as many do not access them. Alternative approaches to outpatient care must be considered, particularly among highly needed services such as orthopedics, in order to support surgical care access within this population. In sum, homeless people’s views and experiences of their own general health, illness symptoms and medical care use are generally associated with their current healthcare utilization.

Having a usual source of care was repeatedly associated with increased service use [25]. However, one may argue that people have usual healthcare providers because they frequently use the service. Gallagher et al. [39] conducted a study in Los Angeles to explore the determinants of having a regular source of healthcare among homeless adults, finding that 57% of the sample had a regular source of care, and the distribution of this characteristic among the homeless was highly inequitable. In Canada, Gentil et al. [31] found that having a family physician was associated with greater user satisfaction among the homeless. Gelberg et al. [6] concluded that better health outcomes were associated with having a community clinic or private physician as a regular source of care. Clinics tailored towards homeless people with highly integrated services were associated with better care experiences among homeless people with serious mental illness [40]. Kertesz et al. [41] found that the tailored design of primary care services was associated with a superior service experience for patients experiencing homelessness. The general physician registrars working at mobile health clinics exhibited decreased negative stereotypes, increased empathy and more knowledge of homeless issues, which could all improve the accessibility of primary care [42]. However, the factor most strongly associated with using the emergency department as a regular source of medical care was a lack of health insurance in the previous year [43]. Having no health insurance or need for care in the previous six months, increased the use of a nonambulatory care site as a place for usual care. Although homeless people are compulsorily enrolled in the National Health Insurance scheme in Taiwan, many homeless people still need to use free outpatient consultation services due to lacking a valid health insurance identity certificate, which is needed in the healthcare system in order to provide alternative or accessible services for this population and thus protect their health rights.

Many factors were found to be associated with high hospital outpatient use through an unadjusted logistical regression model, such as having a usual healthcare provider, having severe depression symptoms, currently taking medicine, having experience of inpatient care and being a chronic disease patient. These factors should be focused on in the healthcare system in order to evaluate the determinants of illness and the most appropriate treatments. The adjusted logistic regression model in this study indicated that homeless people with severe depression symptoms who received medication, and those with chronic diseases, made more free hospital outpatient visits than their counterparts. A systematic review performed by Health Quality Ontario [44] suggested that orienting the clinic services (either alone or combined with outreach) would improve the access of urban homeless adults to a primary care provider. In Dublin, Swabri et al. [35] highlighted the need to expand the healthcare available at mobile health clinics so as to provide homeless people with free and easy access to primary healthcare services, and thus adequately meet the health needs of this target population. Kaplan-Weisman et al. [45] suggested that advance outpatient care planning, even for adults experiencing homelessness, is an effective and valuable use of clinical time, and should be integrated into routine primary care. The present findings illustrate the importance of the needs factors in the Andersen’s model of healthcare utilization; the medical care system needs to monitor the health profiles of homeless people and provide effective medical treatment based on their healthcare needs.

This study is one of the first studies in Taiwan to provide data on the healthcare use of people experiencing homelessness, providing useful information for future health policy formulation. Identifying the factors associated with free outpatient visits can be useful in evaluating the functions of a public health system. The many limitations of this study include its cross-sectional design, the self-report data and the purposive sampling approach, which all prevent the generalization of the results and the establishment of causal relationships between health service use profiles and specific factors among the homeless. Those who do not access outpatient services may not realize that they have health conditions, and those who use more outpatient services may know more about their health and, therefore, use the services more. In addition, the present study only examined hospital-based outpatient service use instead of the whole range of primary healthcare. In future, we might refer to outpatient specialized care for this group of people.

## 5. Conclusions

In this study, we explored the profile of free outpatient care use and its associated factors among middle-aged and elderly homeless people. The results showed that 81.4% of the responding homeless people had made free hospital outpatient visits, and their outpatient care needs were probably higher than those in the general population under the National Health Insurance scheme in Taiwan. The adjusted logistic regression model indicates that homeless people with severe depression symptoms, who are receiving medication and have chronic diseases, made a significantly higher number of free outpatient visits than their counterparts. Policy efforts should address barriers to the use of ambulatory care services, and behavioral health services in particular, to help reduce acute care use and improve the long-term health of homeless individuals [33]. Primary healthcare programs specifically tailored to homeless individuals might be more effective than standard primary healthcare [46]. However, improved healthcare accessibility alone may not be enough. As Reuler [47] stated, the removal of financial barriers to care by the enactment of a national health program in the U.S. would not solve all the issues related to the delivery of quality care for the homeless unless its structure addressed the special needs of disenfranchised groups. It is suggested that these services must achieve integration at the therapeutic and organizational levels so as to better meet the needs of this complex and heterogeneous population [48]. In addition, healthcare must be integrated with other resources to address the complex challenges presented by inadequate housing, hunger and unsafe environments [7].

## Figures and Tables

**Table 1:** Demographic characteristics of the participants (*n* = 129).

Variable	*n* (%)
Sex	
Male	114 (88.4)
Female	15 (11.6)
Age (years, mean ± SD)	58.3 ± 7.5
Level of education	
Elementary or less	36 (27.9)
Junior high school	39 (30.2)
Senior high school	43 (33.3)
College and above	11 (8.6)
Marital status	
Single	66 (51.2)
Married	6 (4.7)
Divorced	47 (36.4)
Other	10 (7.7)
Employment status	
Full-time	18 (14.0)
Part-time	58 (44.9)
None	53 (41.1)
Source of income	
Job earning	71 (55.1)
Allowance for the disabled	15 (11.6)
Subsidy for low income	4 (3.1)
Other	39 (30.2)
Usual healthcare provider	
No	59 (45.7)
Yes	70 (54.3)
Total score of PHQ-9 ^a^ (Mean ± SD)	12.1 ± 6.5
Depression severity ^b^	
No	20 (15.5)
Mild (score 5–9)	21 (16.3)
Moderate (score 10–14)	41 (31.8)
Moderately severe (score 15–19)	34 (26.4)
Severe (score 20–27)	13 (10.1)

^a^ PHQ-9, Patient Health Questionnaire-9. ^b^ Depression severity category (PHQ-9 score): none (0–4), mild (5–9), moderate (10–14), moderately severe (15–19) and severe (20–27).

**Table 2:** Free hospital outpatient visits in the prior three months (*n* = 129).

Medical Department	Number (%); Visits (Mean ± SD)
Free outpatient visits	
Yes	105 (81.4)
No	24 (18.6)
Use of free outpatient visits (Mean ± SD)	5.9 ± 8.1
Frequency of outpatient visits	
<3 (low)	75 (58.1)
≥3 (high)	54 (41.9)
Use of medical department	
Psychiatric	9.14 ± 10.97
Gastroenterology	8.68 ± 10.24
Dermatology	8.63 ± 11.13
Orthopedics	8.48 ± 9.40
Dental	7.91 ± 9.74
Cardiovascular	6.32 ± 4.88
Neurology	5.70 ± 5.78
Other	7.37 ± 8.84

**Table 3:** One-way analysis of free hospital outpatient visits (*n* = 129).

Variables	Use of Free Outpatient Visits ^a^		
	Low; *n* (%)	High; *n* (%)	*p*-Value
Sex			0.204
Male	64 (85.3)	50 (92.6)	
Female	11 (14.7)	4 (7.4)	
Age (years, mean ± SD) *	58.5 ± 7.5	58.2 ± 7.7	0.817
Level of education ^§^			0.878
Elementary or less	19 (25.3)	17 (31.5)	
Junior high school	24 (32.1)	15 (27.8)	
Senior high school	25 (33.3)	18 (33.3)	
College and above	7 (9.3)	4 (7.4)	
Marital Status ^§^			0.690
Single	41 (54.7)	25 (46.3)	
Married	4 (5.3)	2 (3.7)	
Divorced	24 (32.0)	23 (42.6)	
Others	6 (8.0)	4 (7.4)	
Employment status			0.174
Full-time	14 (18.7)	4 (7.4)	
Part-time	33 (44.0)	25 (46.3)	
None	28 (37.3)	25 (46.3)	
Usual healthcare provider			0.006
No	42 (56.0)	17 (31.5)	
Yes	33 (44.0)	37 (68.5)	
Total score of PHQ-9 (mean ± SD) *	10.7 ± 6.2	14.2 ± 6.4	0.002
Depression severity ^b^			0.002
No	16 (21.3)	4 (7.4)	
Mild	10 (13.1)	11 (20.4)	
Moderate	30 (40.1)	11 (20.4)	
Moderately severe	16 (21.3)	18 (33.3)	
Severe	3 (4.0)	10 (18.5)	
Medication within one year			<0.001
No	31 (41.3)	4 (7.4)	
Yes	44 (58.7)	50 (92.6)	
Hospitalized within one year			0.007
No	37 (49.3)	14 (25.9)	
Yes	38 (50.7)	40 (74.1)	
Frequency of outpatient visits (Mean ± SD)	2.4 ± 4.3	11.9 ± 9.5	<0.001
Number of chronic diseases			<0.001
≤3	40 (53.3)	11 (20.4)	
4–5	25 (33.3)	15 (27.8)	
>5	10 (13.4)	28 (51.8)	

^a^ Low: <3 free outpatient visits; high: ≥3 outpatient visits; ^b^ depression severity category (PHQ-9 score): none (0–4), mild (5–9), moderate (10–14), moderately severe (15–19), and severe (20–27); * *t*-test; ^§^ Fisher’s exact test.

**Table 4:** Logistic regression analysis of high level of free hospital outpatient visits (*n* = 129).

Variables	Unadjusted		Adjusted ^a^	
	OR (95 CI%)	*p*-Value	OR (95 CI%)	*p*-Value
Sex, female (ref: male)	0.47 (0.14–1.55)	0.213		
Age (years)	1.04 (0.64–1.69)	0.872		
Level of education (ref: elementary or less)				
Junior high school	0.70 (0.28–1.75)	0.444		
Senior high school	0.81 (0.33–1.96)	0.633		
College and above	0.64 (0.16–2.57)	0.528		
Marital Status (ref: single)				
Married	0.82 (0.14–4.81)	0.826		
Divorced	1.57 (0.74–3.35)	0.242		
Other	1.09 (0.28–4.26)	0.898		
Employment status (ref: no)				
Part-time	0.85 (0.40–1.79)	0.667		
Full-time	0.32 (0.09–1.10)	0.071		
Usual healthcare provider (ref: no)	2.77 (1.33–5.77)	0.006	1.67 (0.62–4.46)	0.309
Depression severity ^§^ (ref: no)				
Mild	4.40 (1.10–17.68)	0.037	4.21 (0.8–20.61)	0.077
Moderate	1.47 (0.40–5.36)	0.562	1.16 (0.25–5.28)	0.853
Moderately severe	4.50 (1.24–16.28)	0.022	8.02 (0.50–11.41)	0.273
Severe	13.33 (2.45–72.45)	0.003	9.32 (1.15–56.07)	0.036
Medication within one year (ref: no)	8.81 (2.88–26.92)	<0.001	3.93 (1.06–14.52)	0.040
Hospitalized within one year (ref: no)	2.78 (1.30–5.94)	0.008	2.06 (0.77–5.52)	0.150
Number of chronic diseases ^§^ (ref: ≤3)				
4–5	2.18 (0.87–5.50)	0.098	1.06 (0.35–3.21)	0.913
>5	10.18 (3.81–27.22)	<0.001	1.06 (1.35–13.27)	0.013

^a^ OR was adjusted by variables that were significant in the unadjusted analysis; ^§^ *p* for trend < 0.05 in unadjusted analysis. OR: odds ratio; CI: confidence interval.

## Data Availability

The data presented in this study are available from the corresponding authors upon reasonable request.
